# A Novel Acoustic Filtering Sensor for Real-Time Tension Monitoring of Hoist Wire Ropes

**DOI:** 10.3390/s18092864

**Published:** 2018-08-30

**Authors:** Xiaoguang Zhang, Zhenyue Song, Jianpu Da, Jianbao Fu

**Affiliations:** School of Mechatronic Engineering, China University of Mining & Technology, No.1 Daxue Road, Xuzhou 221116, China; doctorzxg@163.com (X.Z.); TS14050025@cumt.edu.cn (J.D.); TS16050092P2@cumt.edu.cn (J.F.)

**Keywords:** acoustic filtering sensor, real-time tension monitoring, structural and acoustic parameters, filtering characteristics

## Abstract

The real-time tension monitoring of wire ropes is a universal way to judge whether the hoist is overloaded in the special working environment of the coal mine. However, due to the strong drafts, unevenness of guide and flexible vibration of wire ropes, it is a challenge to monitor the tension with high accuracy. For this purpose, a new type of acoustic filtering sensor is designed in this study. To adapt to the violent vibration during the monitoring process, a structure with a cylindrical cavity and a narrow gap is designed in the sensor. The coupling between the internal fluid and sensor structure can greatly absorb the vibration energy. With the view of optimizing the filtering performance of the sensor, the influences on the filtering characteristics are presented and analyzed through employing different structural and acoustic parameters in simulations. Finally, acoustic filtering sensor prototypes based on optimized parameters are calibrated and tested in a real coal mine. The results have revealed that our acoustic filtering sensor can not only address the deficiencies of current pressure sensors in coal mining and achieve tension monitoring in real-time, but is also able to diagnose and forecast the occurrence of tension imbalance accidents.

## 1. Introduction

As key pieces of equipment for production and transportation in coal mines, hoists, known as the “mine throat”, play a vital role in ensuring production safety [1]. They are commonly used to lift minerals, equipment or transport workers. During their long running process, hoist problems may cause a hazardous incident such as overloading and tension imbalance of the wire ropes [2]. Therefore, the real-time tension monitoring for wire ropes has great theoretical and practical significance to ensure safe production in coal mines.

Nowadays, a variety of methods have been applied to monitor the tension of hoist wire ropes. According to the evaluation criterion of whether the lifting weight is varying during the monitoring process, these methods can be mainly divided into two categories: static monitoring and dynamic monitoring. Static monitoring methods include the flex-cable method [3], bending strains method [4], inhomogeneous chord vibration method [5], etc. With the development of wireless communication technology, dynamic tension monitoring for wire ropes has become a major trend. Dynamic monitoring methods involve the motor current method [6], series connecting sensor method [7,8], oil pressure method [9,10], etc. However, the wire ropes of hoist are flexible and easily subjected to coupled vibration that often leads to serious interferences with the tension signals [11]. In addition, vibration elimination and noise filtering of conventional monitoring methods are mostly performed after data acquisition [5,6,7,8,9,10], so they are hardly suitable for real-time tension monitoring. Thus, it is urgent to design an applicable sensor for the violent vibration environment of coal mines. Many scholars have done a lot of research on similar sensors in the field of industry. In summary, sensors are designed from a variety of aspects to reduce the influence of vibration on measurement results. For instance, in the aspect of sensor materials, the magnetostrictive vibration sensor has several advantages over conventional piezoelectric sensors which are attributed to the high Curie temperature of magnetostrictive materials [12]. In [13], Yang et al. applied a vibration acceleration sensor with the built-in integrated circuit amplifier to adjust vibration signals. Moreover, some studies [14,15] are introduced from the aspect of structure design. With the development of research, the back propagation neural network (BPNN) [16] has been gradually employed to process measurement signals and achieves high accuracy. However, due to poor working conditions and the strict explosion-proof requirements in coal mines, the sensors for tension monitoring are still designed with a focus on sensor structure.

In addition to the approaches mentioned above, at present fluids have satisfactory application prospects in eliminating vibration and absorbing noise in many applications. In [17,18,19], a fluid was applied to reduce the vibration of vehicles. Another typical application of fluid vibration isolation is magneto-rheological fluids (MRF). Nguyen et al. [19] focused on the MRF technology to reduce the noise and vibration transmissibility from the power sources to the vehicle body. In addition, vibration isolators are often applied in the vibration propagation path to protect high precision payloads from vibration disturbances. In [20], on-orbit and launch environment isolation performance of a vibration isolator with a viscous fluid was evaluated. The test results indicated that the developed isolator with viscous fluid provided effective isolation for the small amplitude vibration disturbances and also survived large amplitude vibrations without any damage.

It is worth noting that fluids belong to a kind of dense media, and the interaction between fluid and solid cannot be neglected, namely, the effect of fluid-solid coupling on filtering characteristics needs to be taken into account. Henderson and Miles [21] proposed that the fluid flowing in a container was approximately an ideal flow. They obtained a satisfactory result through adding the damping produced by Stokes dissipation at boundary layers to the damping produced by Rayleigh dissipation in the fluid. Zhou et al. [22] studied the effect of elastic cavity walls on the acoustic characteristics of a water-filled Helmholtz resonator. Theoretical and experimental results showed that the elasticity of cavity walls significantly affected the acoustic characteristics of the Helmholtz resonator and the resonance frequency shifted to a lower frequency domain. To investigate the vibration-reducing mechanism of viscous liquids, Xiang et al. [23] carried out vibration and noise reduction experiments for a double-layered thin-wall box. The results indicated that the increase of box liquid volume and the introduction of diaphragm plates could enhance the damping ratio of the system and reduce the power of acoustic radiation of the system. Griffiths et al. [24] proposed a formula for calculating the filtering frequency of a Helmholtz resonator in consideration of the elastic deformation due to fluid-solid coupling. The research verified that liquid-solid coupling would produce a low noise-absorbing frequency. At the same time, the viscous damping of the fluid played an important role in the vibration energy dissipation.

In view of the situation that the violent disturbance occurs frequently during the tension monitoring phase and some advanced methods are impracticable for existing mining pressure sensors, a novel acoustic filtering sensor based on fluid-solid coupling with a special structure is designed in this paper.

The remainder of this paper is organized as follows: Section 2 introduces the hardware of our tension monitoring system for a multi-rope hoist. Section 3 describes the structure, measuring and filtering principle of the acoustic filtering sensor. Simulation analysis based on fluid-solid coupling is elaborated in Section 4. Section 5 refers to static characteristics tests and an experiment contrasting our acoustic filtering sensor with a universal pressure sensor. Finally, Section 6 concludes the study and discusses some future directions.

## 2. Hardware Description of Tension Monitoring System for a Multi-rope Hoist

**Figure 1:**
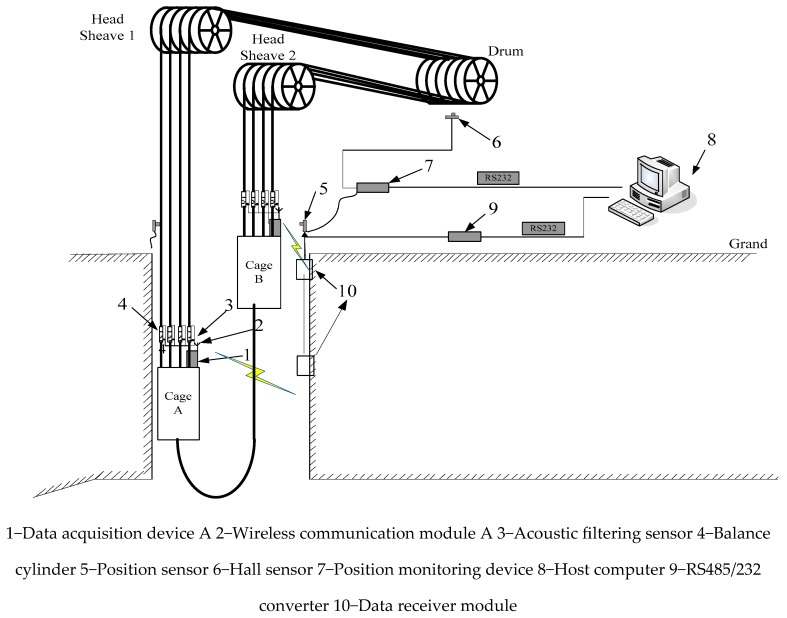
Schematic diagram of tension monitoring system for a multi-rope hoist.

The tension monitoring system for wire ropes of multi-rope hoist is shown in Figure 1. Taking the cage A as an example, the acoustic filtering sensors 3 are installed in the middle of balance cylinders 4. Firstly, the tension signals acquired through data acquisition device 1 are transmitted to the wireless communication module 2. Secondly, the communication module 2 sends the acquired signals to the data receiving module 10 which installed in the well. Then, the signals are transferred to the hoist house by RS485/232 converter (self-made). Finally, the data are processed by host computer 8 to achieve tension monitoring of the wire ropes. In order to illustrate the change regulation of tension with the depth of cage A, cage position signals and cage in position signals are also transmitted to the host computer 8, which are acquired by Hall sensor 6 and position monitoring device 7, respectively.

**Figure 2:**
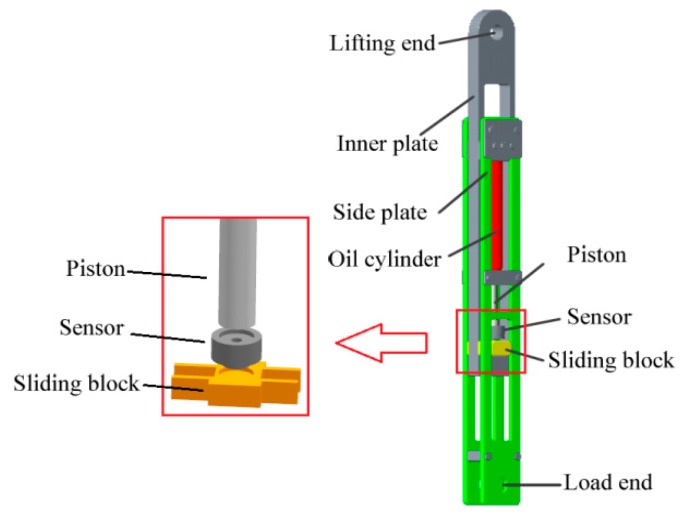
The balance cylinder model of a hoist.

To illustrate the feasibility of our monitoring system, the balance cylinder model of a hoist is established in Figure 2. The model is mainly composed of the inner plate, the side plate, the hydraulic cylinder, the piston rod and the sliding block. The top of inner plate is the lifting end which is connected with one wire rope through a pinhole and the other end is connected with the bottom of sliding block through a groove. The upper end of side plate is connected with the oil cylinder and the bottom is the load end which is connected with the cage through a pinhole. The acoustic filtering sensor is installed between the piston rod and sliding block. In the running process of hoist, the traction acting on the lower end of acoustic filtering sensor is equal to the tension of wire rope, which is denoted as $P_{0}$. The load end acts on the upper end of acoustic filtering sensor through the side plate and the piston rod, which is denoted as $P_{1}$. Under the condition of dynamic load being ignored, $P_{0}=P_{1}$. Consequently, the tension of wire ropes can be obtained based on above monitoring system.

## 3. Sensor Design

### 3.1. Structure of the Sensor

**Figure 3:**
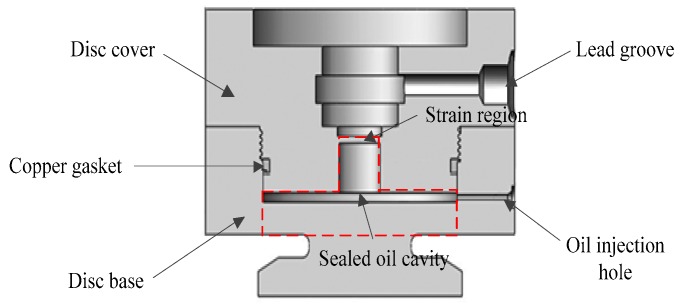
Structure diagram of the acoustic filtering sensor.

Figure 3 demonstrates the structure diagram of the acoustic filtering sensor. The steel-made sensor is mainly composed of a disc cover and a disc base. These two parts are welded after thread connection, which form a cylindrical cavity and a flat gap. We select dimethylsilicone oil as the fluid filling in the sensor. To prevent oil leakage, a gasket is installed at the joint part of disc cover and disc base. Considering that the gasket will not be replaced again once the sensor is welded, copper is selected as the gasket material. Strain region, a circular thin plate, is set in the center of the disc cover, and strain gauges are attached on the upper side of strain region to measure oil pressure. We also provide a lead groove in the middle of disc cover for wiring and protecting ropes. Furthermore, an oil injection hole is designed in the lower part of disc base to inject oil into the cavity and gap.

To prevent air from entering the oil cavity, oil injection needs to be accomplished with a special vacuum pumping device. 

**Figure 4:**
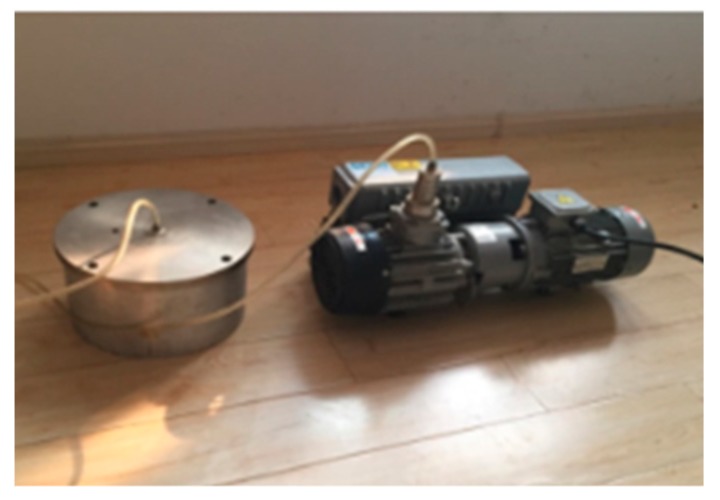
The device for pumping vacuum.

As shown in Figure 4, the device in the left is used to contain dimethylsilicone oil and it connects with the right vacuum pump through a plastic pipe. The oilless sensor is immersed in the oil and then the vacuum pump begins to pump the air remaining in the container. With the decrease of internal pressure, the air-free oil is injected into the sensor gradually through the oil injection hole. Finally, the hole is sealed with a screw in the oil after oil injection.

### 3.2. Measuring Principle of the Sensor

**Figure 5:**
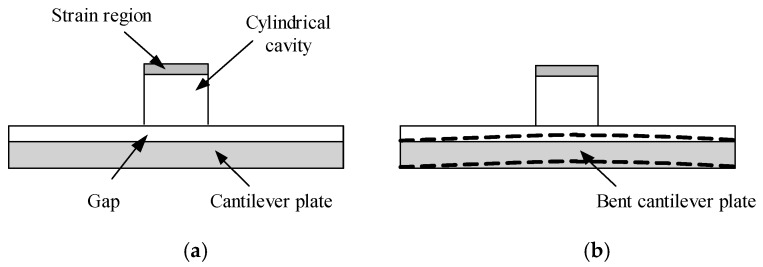
Schematic of measuring principle of acoustic filtering sensor. (**a**) The sensor structure under normal conditions; (**b**) The sensor structure when being squeezed and bent.

According to the description in Section 2, an external load acts on the disc cover of the sensor through balance cylinder, and the traction of wire ropes acts on the disc base of the sensor. Under the action of these two forces, the sensor will be squeezed and bent. The deformation mainly occurs in the red area of Figure 3. To search the relationship between traction and external load, we simplify the tagged sensor structure as Figure 5a. The lower circular thin plate in red area is equivalent to a cantilever plate from the aspect of mechanics. The cantilever plate will be bent as Figure 5b when confronting with external forces, changing the oil cavity volume and internal oil pressure. Consequently, the increased oil pressure results in the deformation of strain region and measured output value is capable of calculating the tension of wire ropes.

For better understanding the relationship between oil cavity volume and oil pressure, the oil compressibility coefficient *β* is introduced. Assuming the change of oil cavity volume is slight and the velocity of sound in oil fluid is a constant, the oil pressure *p*_0_ can be deduced by Equation (1):

(1) $$p_{0}=-\frac{1}{\beta }\frac{\Delta V}{V}=-\frac{\rho_{0}c_{0}^{2}\Delta V}{V},$$

where ΔV represents the changed volume of oil cavity, *V* represents the initial volume of oil cavity, $\rho_{0}$ is the oil density, and $c_{0}$ is the velocity of sound in oil fluid. In the case of small disturbance, $c_{0}^{2}=1/\beta \rho_{0}$.

The volume change of oil cavity is originated from two factors: the deformation of the cantilever plate and the deformation of the strain region. The cantilever plate is subjected to an upward counterforce from the disc base when an external load *p* acts on the disc cover. We assume that the counterforce acts on the whole face of cantilever plate for simplifying the calculation. At this time, the question is equivalent to the bending of circular thin plate under uniform load. The bending displacement of circular thin plate under static loading can be expressed as follows [22]:

(2) $$\omega (r)=\frac{pa^{4}}{64D}{\left(1-\frac{r^{2}}{a^{2}}\right)}^{2},$$

where *p* denotes the uniform load acting on circular thin plate, *a* denotes the radius of circular thin plate, *D* is the anti-bending rigidity of circular thin plate, and *r* is the polar radius.

The volume change produced by the displacement of circular thin plate can be described as:

(3) $$\Delta V=\int_{0}^{a}2\pi r\cdot \omega (r)dr=\int_{0}^{a}2\pi r\frac{pa^{4}\;}{64D}{\left(1-\frac{r^{2}}{a^{2}}\right)}^{2}dr=\frac{\pi pa^{2}}{192D}.$$

Based on Equations (2) and (3), when the sensor is in a steady state, the bending displacement and volume change of cantilever plate can be expressed as follows:

(4) $$\left\{ \begin{array}{l} \omega_{1}(r)=\frac{(p-p_{0})a_{1}^{4}}{64D_{1}}(1-\frac{r^{2}}{a_{1}^{2}}{)}^{2}\\ \Delta V_{1}=\frac{\pi (p-p_{0})a_{1}^{2}}{192D_{1}} \end{array}\right..$$

The bending displacement and volume change of strain region under the action of oil pressure are described in a similar way, that is:

(5) $$\left\{ \begin{array}{l} \omega_{2}(r)=\frac{p_{0}a_{2}^{4}\;}{64D_{2}}(1-\frac{r^{2}}{a_{2}^{2}}{)}^{2}\\ \Delta V_{2}=\frac{\pi p_{0}a_{2}^{2}}{192D_{2}} \end{array}\right.,$$

where $a_{1}$ and $a_{2}$ are the radius of cantilever plate and strain region, $D_{1}$ and $D_{2}$ are the anti-bending rigidity of cantilever plate and strain region, respectively.

Thus, the volume change of oil cavity under the action of external load *p* can be deduced as follows:

(6) $$\Delta V=\Delta V_{1}-\Delta V_{2}=\frac{\pi (p-p_{0})a_{1}^{2}}{192D_{1}}-\frac{\pi p_{0}a_{2}^{2}}{192D_{2}}.$$

Based on Equations (1), (4), (5) and (6), the oil pressure and the volume change of cantilever plate and strain region under the action of external load can be written as:

(7) $$p_{0}=\frac{a_{1}^{6}}{\frac{192D_{1}V}{\rho_{0}c_{0}^{2}\pi }+a_{1}^{6}+\frac{D_{1}}{D_{2}}a_{2}^{6}}p,$$

(8) $$\Delta V_{1}=\pi \frac{(\frac{a_{2}^{6}\;}{192D_{2}}+\frac{V}{\rho_{0}c_{0}^{2}\pi })a_{1}^{6}}{\frac{192D_{1}V}{\rho_{0}c_{0}^{2}\pi }+a_{1}^{6}+\frac{D_{1}}{D_{2}}a_{2}^{6}}p,$$

(9) $$\Delta V_{2}=\pi \frac{\frac{a_{1}^{6}a_{2}^{6}}{192D_{2}}}{\frac{192D_{1}V}{\rho_{0}c_{0}^{2}\pi }+a_{1}^{6}+\frac{D_{1}}{D_{2}}a_{2}^{6}}p.$$

Other specific parameters of the acoustic filtering sensor are shown in Table 1. Based on these parameters, the oil cavity volume is $V\approx \pi \times (30^{2}\times 2+7^{2}\times 10)=2290\pi \;{\mathrm{mm}}^{3}$, the anti-bending rigidity of cantilever plate is $D_{1}=6470\;N\cdot m^{2}$, and the anti-bending rigidity of strain region is $D_{2}=18.86\;N\cdot m^{2}$. Substituting these calculations into Equations (8) and (9), we obtained $\Delta V_{2}\approx 0.022\Delta V_{1}$. Due to the tiny value of $\Delta V_{2}$, the effect of strain region deformation on the volume change of oil cavity can be neglected, namely, the volume change of oil cavity is merely caused by the deformation of cantilever plate.

As a result, $p_{0}$ can be rewritten as:

(10) $$p_{0}=-\frac{\rho_{0}c_{0}^{2}\Delta V}{V}=\frac{\rho_{0}c_{0}^{2}(\Delta V_{1}-\Delta V_{2})}{V}\approx \frac{\rho_{0}c_{0}^{2}\Delta V_{1}}{V}.$$

Once the size of the acoustic filtering sensor and the type of internal oil are determined, $\rho_{0}c_{0}^{2}/V$ becomes a constant. According to Equation (10), there is a linear relationship between $p_{0}$ and $\Delta V_{1}$. Similarly, according to Equation (8), there is a linear relationship between $\Delta V_{1}$ and external load *p*. Thus, the internal oil pressure $p_{0}$ varies linearly with the external load *p*. This is the measurement principle of the acoustic filtering sensor for tension monitoring of wire ropes.

### 3.3. Filtering Principle of the Sensor

The fluid in oil cavity will be squeezed and it will vibrate when the cantilever plate is bent. The oil cavity is composed of two parts: a gap and a cylindrical cavity. Assuming the fluid mass in the gap is $M_{1}$ and the fluid mass in the cylindrical cavity is $M_{2}$, due to the narrow width of the gap, the energy of $M_{1}$ is dissipated through the gap damping. In addition, resonance will occur if the parameters are designed to make the vibration frequency closed to the natural frequency of fluid $M_{1}$. By this way, the kinetic energy contained in fluid $M_{1}$ is dissipated to the maximum. As a result, the vibration of fluid $M_{2}$ will be weakened so that the oil pressure acting on strain region and output voltage signals generated by strain gauges become more stable.

**Figure 6:**
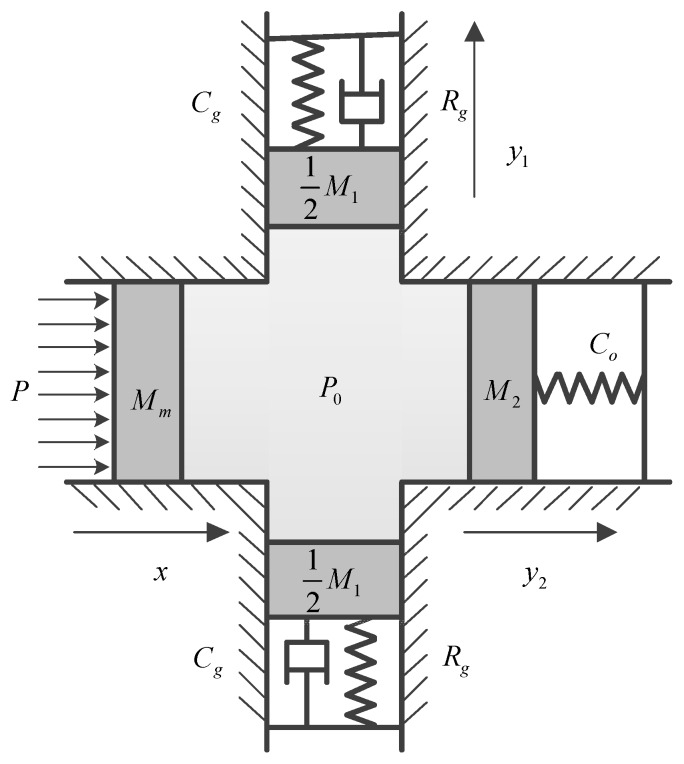
The equivalent vibration model of oil cavity.

In order to illustrate the filtering principle of acoustic filtering sensor, the dynamics model of oil cavity is established. The gap is flat, narrow and its volume is larger compared with the cylindrical cavity, which produces damping effect and elasticity effect when fluid $M_{1}$ flows in the gap. Therefore, the gap can be simplified as a spring-mass-damp system. For the fluid $M_{2}$, the strain region will be squeezed when it flows in the cylindrical cavity, and the effect of strain region on fluid $M_{2}$ is equivalent to a spring at this time. Therefore, the cylindrical cavity can be simplified as a spring-mass system. Figure 6 presents the equivalent vibration model of oil cavity.

In order to simulate the excitation force acting on cantilever plate, the motion of this circular thin plate can be regarded as the vibration of piston, and its equivalent mass is denoted as $M_{m}$. Assuming the displacements of fluid $M_{m}$, $M_{1}$ and $M_{2}$ are x, $y_{1}$ and $y_{2}$, respectively; the equivalent acoustic area of fluid $M_{m}$, $M_{1}$ and $M_{2}$ are $S_{0}$, $S_{1}$ and $S_{2}$, respectively.

For the cantilever plate, its kinetic equation is:

(11) $$(p-p_{0})S_{0}=M_{m}\ddot{x}.$$

For fluid $M_{1}$ flowed in the gap, its kinetic equation is:

(12) $$p_{0}S_{1}=\frac{1}{2}M_{1}{\ddot{y}}_{1}+R_{g}{\dot{y}}_{1}+\frac{1}{C_{g}}y_{1},$$

where $R_{g}$ is the acoustic resistance of the gap, $C_{g}$ is the acoustic conductor of the gap.

For fluid $M_{2}$ flowed in the cylindrical cavity, its kinetic equation is:

(13) $$p_{0}S_{2}=M_{2}{\ddot{y}}_{2}+\frac{1}{C_{0}\;}y_{2},$$

where $C_{0}$ is the acoustic conductor of the cylindrical cavity.

Assuming the fluid is in an ideal state of adiabatic compression and substituting $S_{0}$, $S_{1}$, $S_{2}$, x, $y_{1}$ and $y_{2}$ into Equation (1), the oil pressure can be obtained as:

(14) $$p_{0}=-\frac{\rho_{0}c_{0}^{2}\;}{V}(S_{0}x-2S_{1}y_{1}-S_{2}y_{2}).$$

As shown in Figure 6, the fluid $M_{1}$, fluid $M_{2}$, gap and cylindrical cavity together constitute a dynamic vibration absorbing system. Fluid $M_{1}$ will resonate when the noise frequency is equal or close to its natural frequency. Then, the vibration energy of fluid $M_{1}$ is real-timely absorbed under the action of gap damping. Thus, the vibration amplitude of the other vibration absorber will be reduced, namely, the oil pressure acting on the strain region becomes more stable. This is the real-time filtering principle of the acoustic filtering sensor.

## 4. Simulation Analysis of the Sensor

### 4.1. Effect of Fluid-Solid Coupling on Natural Frequency of the Sensor

**Figure 7:**
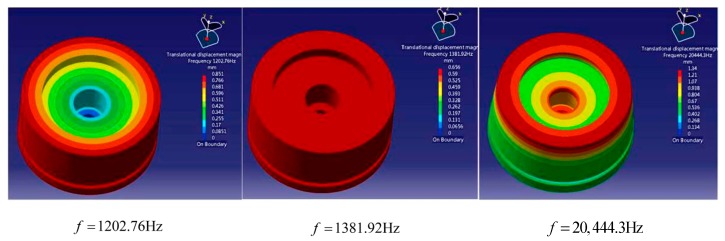
The first three order modal graphs of the sensor without fluid in the cavity.

Based on the analysis in Section 3.3, we conclude that the sensor can filter out the noise signals through the interaction between sensor shell and internal fluid. However, the natural frequency of structure will be changed in consideration of fluid-solid coupling [25]. Therefore, to study the effect of internal fluid on natural frequency of the sensor, modeling and simulation for the designed sensor are conducted in LMS Virtual Lab/Acoustic environment under the condition of the cavity being vacuumed and filling with different fluids. The signal frequency ranges from 0 to 20 kHz in this study. We select the first three order modals as the analysis object. The modal graphs of the sensor when sealed cavity is vacuumed are shown in Figure 7. On the basis of theoretical calculation, the probable natural frequency of the established 3D model is 1380 Hz. We gradually increase the excitation frequency on this foundation until reaching 20 kHz. As seen from the following modal graphs, the deeper the color is, the more intense the vibration is. The second order modal is reddened absolutely, namely, resonance occurs in the sensor. Thus, the frequency of the second order modal is corresponding to the natural frequency of the sensor.

**Figure 8:**
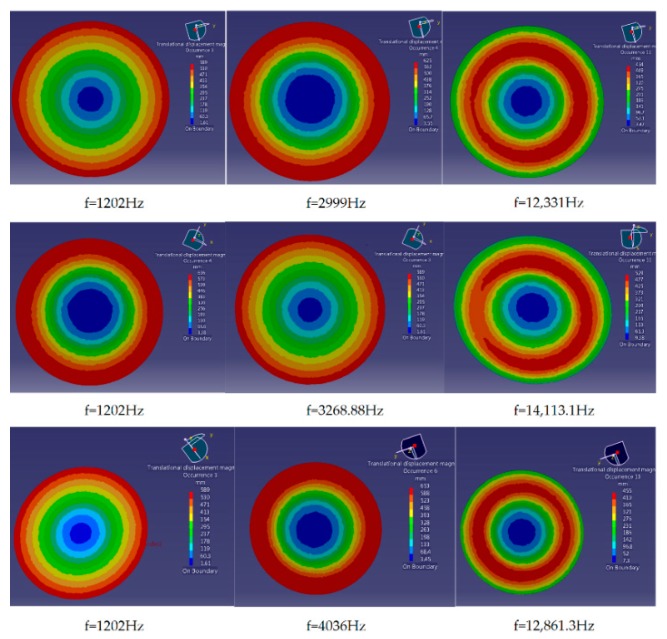
The first three order coupling modal graphs of the sensor filling with different fluids.

Figure 8 illustrates the first three order coupling modal when filling different fluids into the sensor. Lines 1–3 correspond to the coupling modals of the sensor filling with dimethylsilicone oil, water, and mercury, respectively. Compared with Figure 7, the natural frequency of sensor structure has been changed generally, and the natural frequency of the second order coupling modal increased obviously. The reason is that internal oil hinders the bending of cantilever plate and increases the stiffness of sensor structure when the sensor is squeezed. As a result, the effect of fluid-solid coupling on the sensor is presented as the increase of natural frequency.

Table 2 shows the natural frequencies of different orders coupling modals. When the sensor is filled with different fluids, the natural frequencies of the 4th order coupling modal are almost similar, and they approximately equal to 20,443 Hz. When the sensor is filled with neither fluid nor air, the natural frequency of the 3rd order coupling modal also approximately equals to 20,443 Hz. The result indicates that when the sensor is vacuumed, fluid-solid coupling will not change the frequency which is larger than the one of the 3rd order coupling modal. That is, the frequency of the 4th coupling modal remains unchanged. Through analysis, the frequency of the 4th coupling modal is exactly in accordance with the frequency of the thin plate in strain region. This illustrates the resonance characteristics of strain region are irrelevant to the fluid in oil cavity. Therefore, for eliminating the influence of vibration on measurement signals, we merely need to research and control the influence of external disturbance on the internal sound filed of the fluid.

In addition, it is also known from Table 2 that a new natural frequency appears between the 2nd and the 3rd order natural frequencies under the effect of fluid-solid coupling, which indicates that the fluid flowing in oil cavity produces another resonant region and changes the amplitude-frequency characteristics of the sensor. Therefore, to realize better filtering performance, these two resonance regions are to be optimized. On the one hand, the difference between natural frequencies of two resonance regions should be enlarged via reasonable structural and acoustic parameters. On the other hand, the amplitude-frequency characteristics in pivotal frequency bands should be improved via reasonable structural and acoustic parameters.

### 4.2. Effect of Different Parameters on Filtering Characteristics of the Sensor

**Figure 9:**
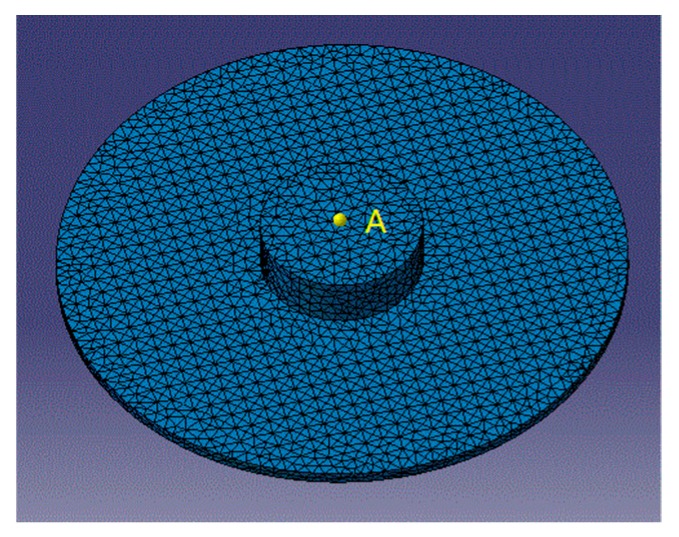
Field point A in fluid.

Structural and acoustic parameters play a significant role in improving filtering characteristics of the sensor. Structural parameters include oil cavity radius, gap width and structural damping. Acoustic parameters include fluid type and acoustic damping. In this subsection, the influence of different parameters on the filtering characteristics is studied by means of controlling variables. As shown in Figure 9, we select a single point A at the center of acoustic envelope surface as the research object. Because point A is under the strain region, its acoustic pressure can approximately reflect the vibration response of the thin plate in strain region.

#### 4.2.1. Effect of Cylindrical Cavity Radius on Acoustic Filtering Characteristics of the Sensor

**Figure 10:**
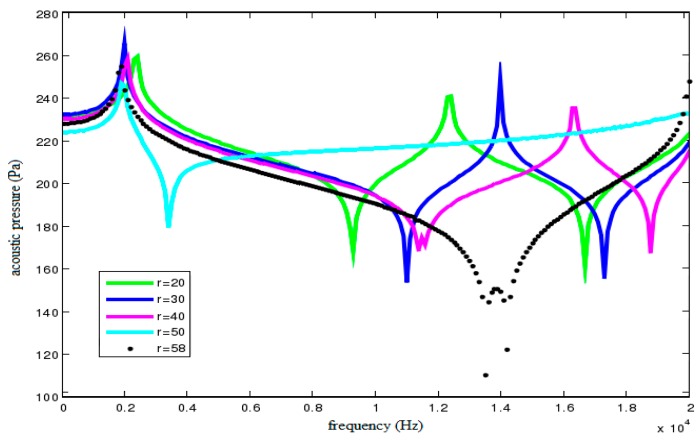
Acoustic pressure characteristic curves of field point A under different cavity radii.

To verify the effect of cylindrical cavity radius on acoustic filtering characteristics of the sensor, a set of radii are tried, *r* = 20, 30, 40, 50 and 58 mm, respectively. An external load of 5 MPa whose frequency ranges from 0 to 20 kHz is applied on the disc cover. Figure 10 shows the acoustic pressure characteristic curves of field point A under different cavity radii when dimethylsilicone oil is filled into the oil cavity.

Combining with the conclusions in Section 4.1, we conclude that the vibration frequency should be in the interval between natural frequencies of the 1st and 2nd order coupling modal, which corresponds to the vibration attenuation region. Therefore, the purpose for reducing the frequency of the 1st order coupling order and increasing the frequency of the 2nd order coupling modal is feasible. As shown in Figure 10, with the increase of cavity radius, the 1st order resonance frequency of acoustic filtering sensor basically remains unchanged whereas the change for the 2nd order resonance frequency is obvious. In addition, the filtering band is gradually widened. However, the peak difference of acoustic pressure curve, namely, the attenuation of vibration is firstly increased and then declined with the increase of cavity radius. As a result, reasonable increase of cavity radius can effectively improve the filtering performance of the sensor.

#### 4.2.2. Effect of Damping on Acoustic Filtering Characteristics of the Sensor

Damping is critical to the noise filtering and vibration absorbing of acoustic filtering sensor. Damping can be divided into structural damping and acoustic damping. The structural damping is produced due to the flat gap and the acoustic damping is produced due to the coupling between oil fluid and sensor shell. As a result, the effect of damping on filtering characteristics of the sensor is investigated from these two aspects. 

**Figure 11:**
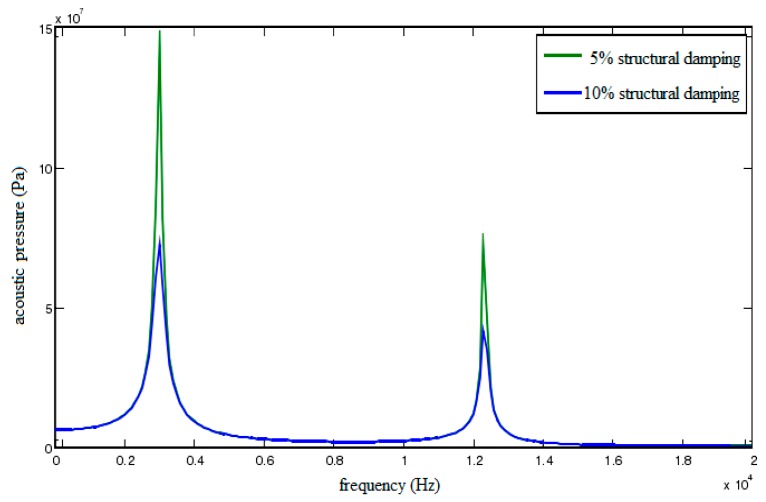
Acoustic pressure characteristic curves of field point A under different structural dampings.

Figure 11 shows the acoustic pressure characteristic curves of point A when structural damping is set as 5% and 10%, respectively. As shown in the figure, not only is the peak of the first resonance region reduced, but the one of the second resonance region is reduced too. Consequently, reasonable structural damping could effectively reduce vibration peaks.

**Figure 12:**
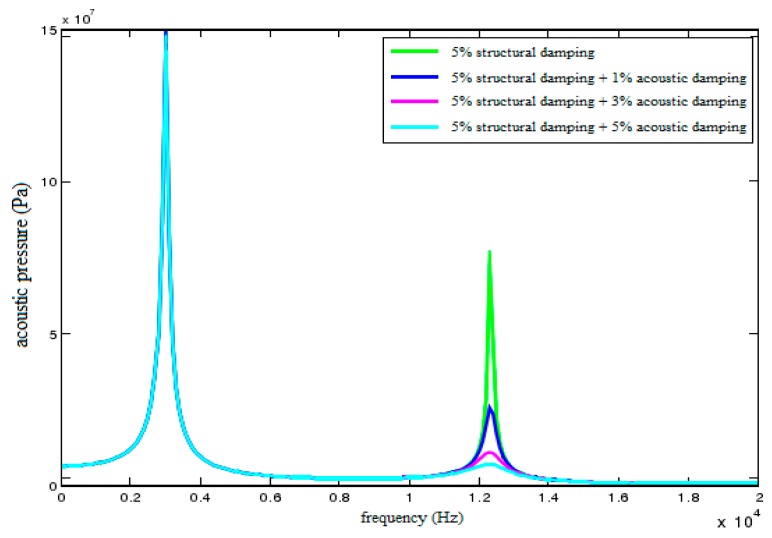
Acoustic pressure characteristic curves of field point A under different acoustic dampings.

In this paper, the effect of fluid viscosity on vibration energy consumption can be regarded as the influence of acoustic damping on the filtering characteristics of the sensor. Here, acoustic damping is set as 0%, 1%, 3%, 5%, respectively. Figure 12 shows the acoustic pressure characteristic curves of field point A under different acoustic dampings. As shown in the figure, the peak of the second resonance region is reduced resulting from the acoustic damping and the effect is more obvious with the increase of the acoustic damping. However, the reduction effect of acoustic damping on the first resonance region is almost negligible.

In Table 2, a new resonance frequency appears between the original 1st and 2nd order frequency after filling the sensor with fluid. That is, an extra resonance region is added for the sensor. The comparison result indicates that the produced second resonance region results from the coupling between the sensor shell and internal fluid. The filtering characteristics of this resonance region are determined by these two factors. However, the filtering characteristics of the first resonance region are only determined by structural damping. Therefore, to achieve optimal filtering performance, it is advisable to reduce the peaks of both resonance regions via setting appropriate structural damping firstly, and then reduce the peak of the second resonance region via setting appropriate acoustic damping.

#### 4.2.3. Effect of Gap Width on Acoustic Filtering Characteristics of the Sensor

**Figure 13:**
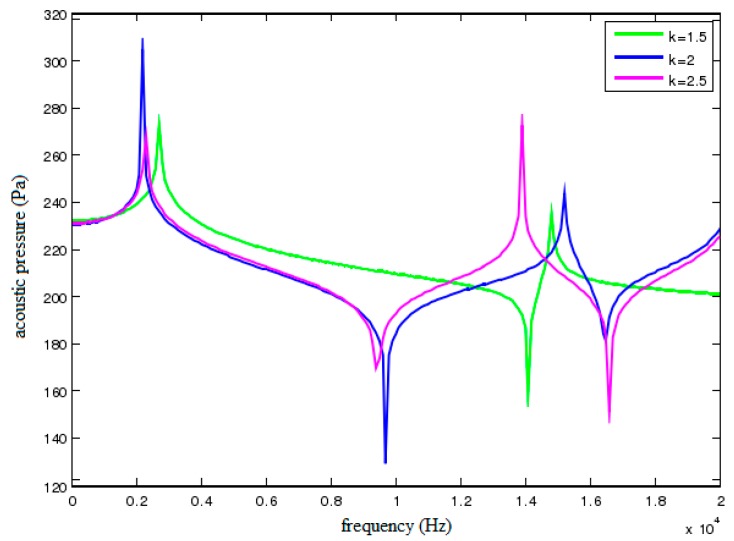
Acoustic pressure characteristic curves of field point A under different gap widths.

The gap has an ingenious effect on the acoustic and structural damping. The acoustic damping is produced when fluid flows in the gap and structural damping is produced resulting from the friction between viscous fluid and solid boundary. Figure 13 shows the acoustic pressure characteristic curves of field point A when the gap width *k* is set as 1.5, 2 and 2.5 mm, respectively. As shown in the figure, the width of filtering band will decrease with the increase of gap width, whereas the volume of oil cavity will increase at the same time. In this case, the additional mass of circular thin plates will also increase, which reduces the natural frequency of the first resonance region. But conversely, the attenuation degree in corresponding filtering band will rise with the increase of gap width. Therefore, the relationship between gap width and filtering characteristics should be balanced to achieve the optimal filtering performance.

#### 4.2.4. Effect of Fluid Media on Acoustic Filtering Characteristics of the Sensor

**Figure 14:**
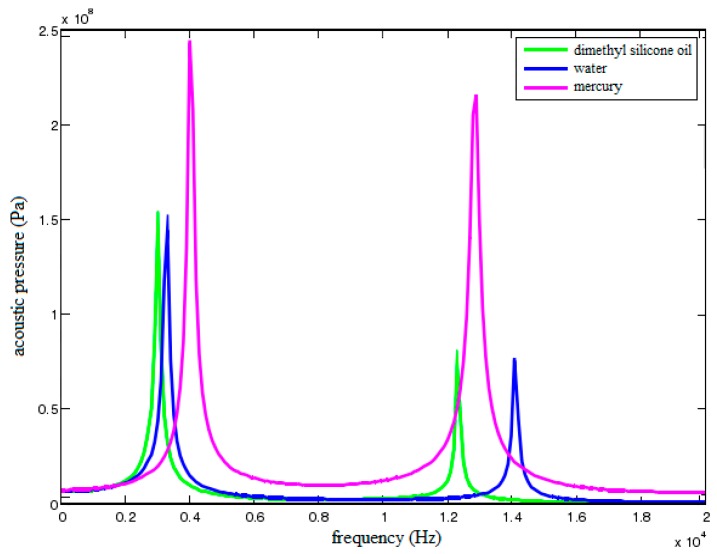
Acoustic pressure characteristic curves of field point A filling with different fluids.

In order to compare the effect of different fluid media on acoustic filtering characteristics of the sensor, three different fluids are filled into the sensor, respectively. The cavity radius is set as 30 mm, the structural damping is set as 5%, the acoustic damping is set as 0%, and the gap width is set as 2 mm. Figure 14 shows the acoustic pressure characteristic curves of filed point A when dimethylsilicone oil, water and mercury are filled into the sensor, respectively.

As shown in the figure, the filtering band of mercury is relatively narrow and its noise attenuation effect is the weakest. The filtering band of water is wider whereas its expansion coefficient is larger than the one of sensor material. Accordingly, additional stress and deformation will be produced due to different expansion degrees when environmental temperature changes, resulting in inaccurate measurement results. For dimethylsilicone oil, the temperature rise coefficient is relatively small and its kinetic viscosity can be adjusted according to actual demand. Therefore, dimethylsilicone oil with suitable kinetic viscosity is helpful to improve acoustic filtering performance of the sensor.

## 5. Performance and Experimental Data Analysis of the Sensor

Based on the simulation results in Section 4, the influence of different structural and acoustic parameters on filtering characteristics of the sensor is obtained. These results play an important role in guiding the design of the sensor. In this section, the optimized structural and acoustic parameters are adopted to manufacture sensor prototypes. Static characteristics tests of the prototypes were performed in the laboratory. In addition, corresponding dynamic experiment was carried out in coal mine.

### 5.1. Static Characteristics of the Sensor

The calibration experiments are necessary for the sensors before their practical engineering applications [15]. The purpose of the calibration for the sensor in this paper is to obtain the relation between the input physical parameter and the output parameter, that is, tension and voltage. In this subsection, static characteristics of the sensor are verified.

**Figure 15:**
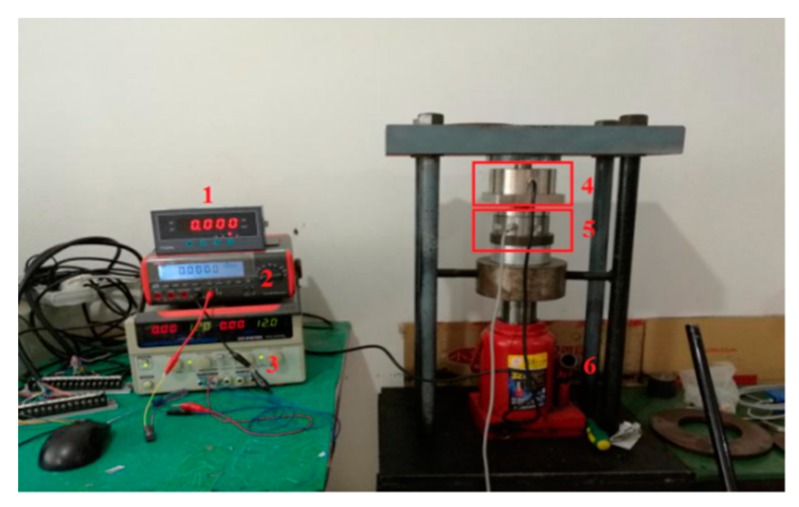
Experimental setup. (**1**) Displayer 1; (**2**) Displayer 2; (**3**) DC power; (**4**) Acoustic filtering sensor; (**5**) Standard sensor; (**6**) Hydro-cylinder.

As shown in Figure 15, the experimental setup is composed of the displayers, the DC power, the acoustic filtering sensor, the standard sensor and the hydro-cylinder. An acoustic filtering sensor is concentrically placed on the standard sensor, and two kinds of sensors are stalled between the hydro-cylinder and bracket. The hydro-cylinder is used to simulate external load. The force of the hydro-cylinder acting on the standard sensor is equal with the one of standard sensors acting on the acoustic filtering sensor. Therefore, it is feasible to verify the linearity via comparing the output voltages of two sensors. In theory, the output voltage should be proportional to external load within measuring range.

**Figure 16:**
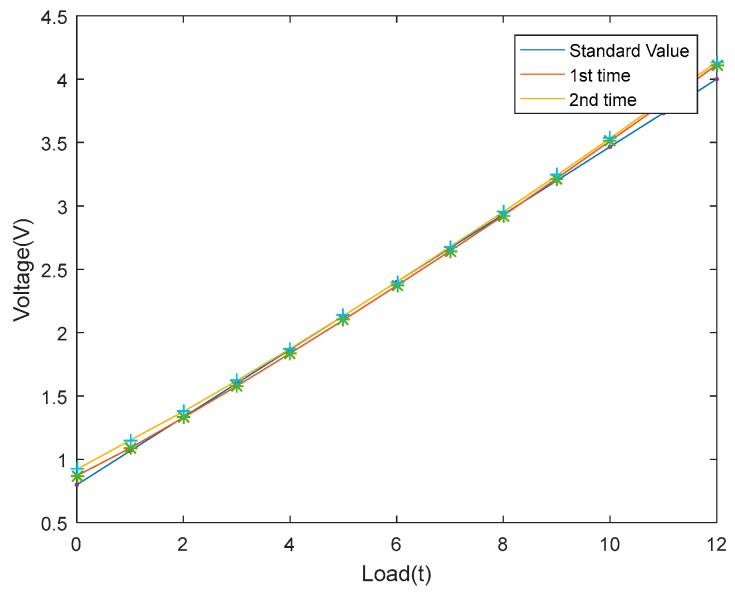
Linearity experiment results of the sensor.

In order to ensure that the linearity and stability of the sensor are credible, a series of experiments were conducted. The first step was to exert the static preload of hydro-cylinder on the standard sensor at room temperature. The preload ranged from 0 to 12 t. Experimental data were recorded when the preload reached an integer each time and the experiment was repeated twice. Standard and measured values were figured in Figure 16. As can be seen, the acoustic filtering sensor has an acceptable linearity.

**Figure 17:**
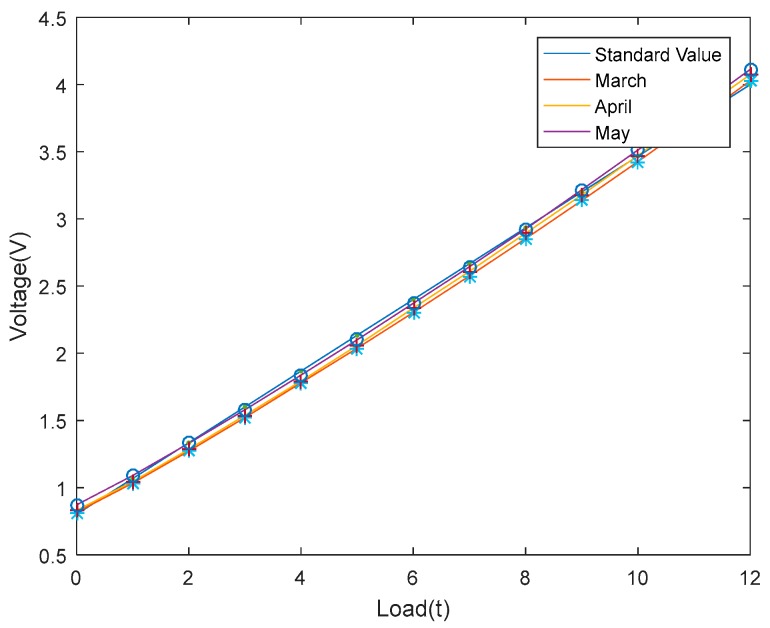
Stability experiment results of the sensor.

Moreover, to verify the stability of the sensor, the same experiment was carried out at three different times. The average output voltages of three times was selected as the final measured result. As shown in Figure 17, the data measured in March, April, May are almost coincident with the data of standard sensor. Thus, the stability of designed sensor is satisfactory.

On the basis of above static characteristics tests, major static indexes of the acoustic filtering sensor meet the requirements of standard sensor. Consequently, it is feasible to apply the acoustic filtering sensor in practical industry.

### 5.2. Dynamic Experiment of the Sensor

**Figure 18:**
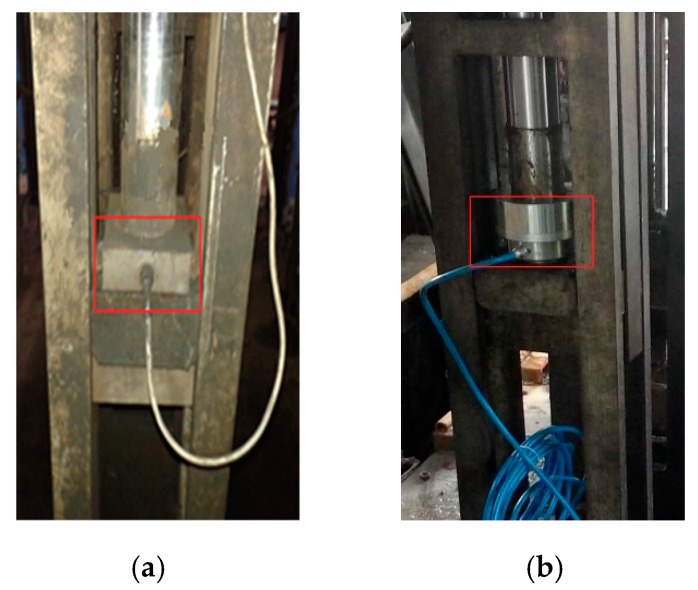
Installation situation. (**a**) Universal pressure sensor; (**b**) Acoustic filtering sensor.

To verify the practical filtering performance of designed sensor, dynamic tension monitoring experiment of wire ropes was carried out in Gao Zhuang coal mine of Shandong Province. Testers installed a universal pressure sensor without any vibration absorption facilities and an acoustic filtering sensor in the same balance cylinder, respectively. The actual installation situation is shown in Figure 18.

**Figure 19:**
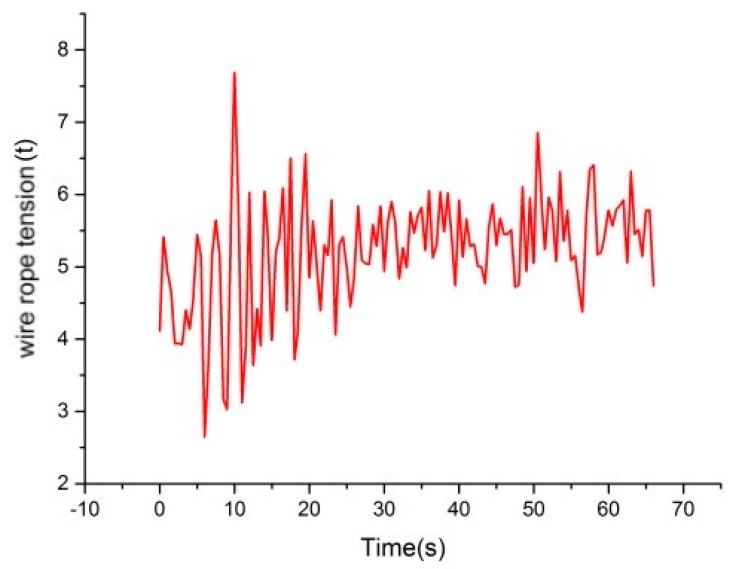
Data of universal pressure sensor.

Taking the lifting process of one cage as an example, tension signals were transmitted steadily without interference. Figure 19 demonstrates the tension measurement result of one wire rope by the universal pressure sensor. As shown in the figure, the tension signals fluctuate violently and the difference of maximal and minimal values even reaches 5 t, whereas the loadage of a cage generally is merely 10 t. Therefore, the sensor is not able to eliminate the influence of vibration on the measured signals. In other words, it is not proposed to monitor the tension of wire ropes through universal pressure sensors.

**Figure 20:**
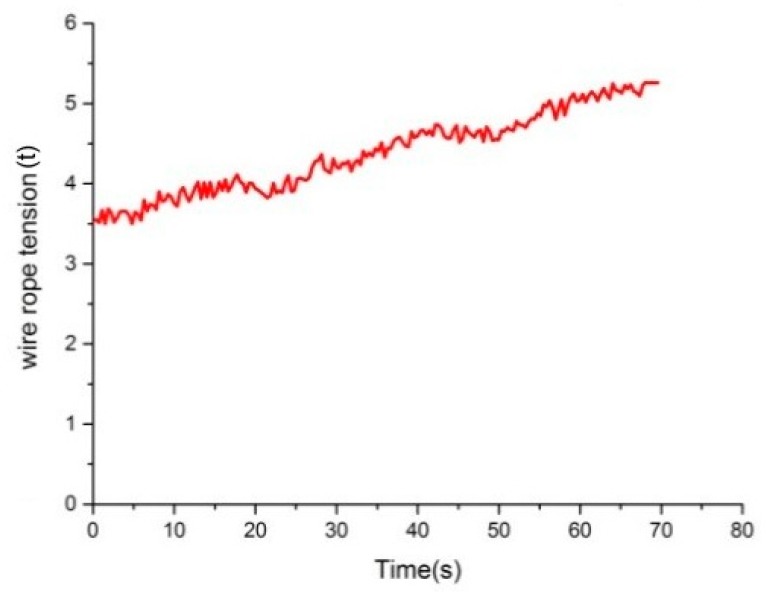
Data of acoustic filtering sensor.

Figure 20 represents the tension measurement result of the same wire rope by acoustic filtering sensor. As shown in the figure, the disturbance to acquired data can be effectively eliminated, and the tension varies with the lifting time in a linear manner approximately. It is also worth mentioning that for the hoist in the Gao Zhuang coal mine, the weight of two tail ropes is about 6 t. In Figure 20, the maximum value of tension difference for one wire rope is about 1.47 t during the whole lifting process. Thus, the summed tension of four wire ropes is about G=1.47×4=5.88t, which is approximately equal to the weight of the tail ropes. The calculation result indicates that signals measured by the designed sensor are reliable. As a result, the acoustic filtering sensor can be applied for dynamic tension monitoring of wire ropes.

**Figure 21:**
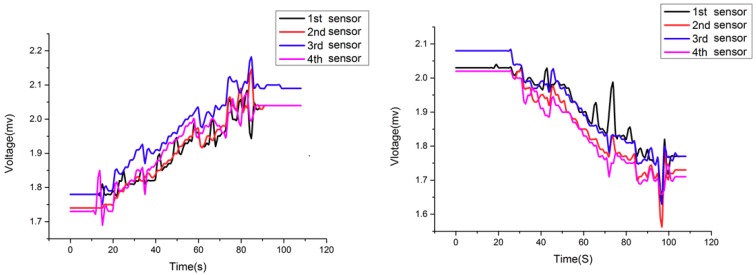
Tension curves of four wire ropes under normal condition.

In addition to measuring the tension of wire ropes, the sensor can also be used to diagnose and forecast the occurrence of tension imbalance accidents. Figure 21 shows the variation of output voltage with ascending and descending time when the balanced suspension device is under normal conditions. As shown in the figure, the difference between the tension of the four wire ropes is tiny. In fact, the fluctuation in the initial and final position is caused by the acceleration and deceleration during the corresponding period. Besides, some slight fluctuation in intermediate process is due to the unevenness of the cage guide. In general, the tension of each wire rope varies linearly with ascending or descending time.

**Figure 22:**
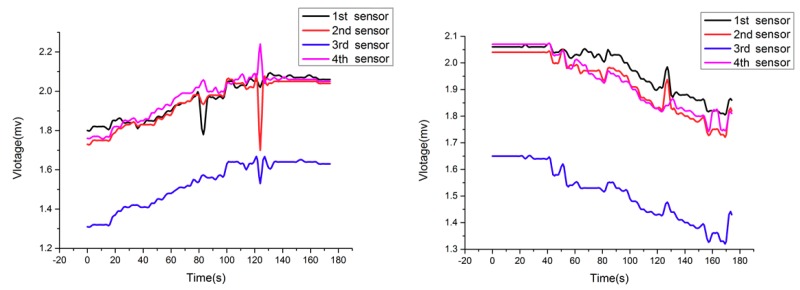
Tension curves of four wire ropes when a valve is closed.

Figure 22 shows the tension of four wire ropes when the valve of the 3rd oil cylinder is closed. As shown in the Figure, the output voltage of the third sensor is obviously lower than other three sensors’. The reason for this phenomenon is that the third oil cylinder lost its balance adjustment ability and the corresponding wire rope is relatively slack at this moment. As a result, the tension of the other three wire ropes is larger than that under normal conditions because they shared the reduced force together.

**Figure 23:**
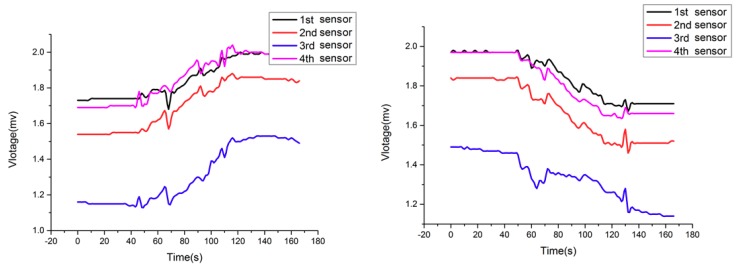
Tension curves of four wire ropes when two valves are closed.

Figure 23 shows the tension of each wire rope when the valves of the 2nd and 3rd oil cylinders are closed at the same time. Similarly, the output voltages acquired by the 2nd and 3rd sensors are obviously different from the other two sensors’.

Thus, the acoustic filtering sensor can not only realize real-time tension monitoring, it is also advisable to apply the sensor for diagnosing and forecasting the occurrence of tension imbalance accidents.

## 6. Conclusions and Future Work

This paper presents an acoustic filtering sensor for real-time tension monitoring of hoist wire ropes. The measuring and filtering principle of the sensor are analyzed through establishment of an equivalent model and theoretical derivation. Moreover, the effect of different structural and acoustic parameters on filtering characteristics is obtained through simulation analysis. In contrast to universal pressure sensors, the acoustic filtering sensor shows better vibration elimination and noise filtering characteristics, and can be further extended for diagnosing and forecasting the occurrence of the tension imbalance accidents. In future studies, the authors plan to investigate quantitatively the effect of different structural and acoustic parameters on filtering characteristics. Possible improvements may include the research on optimal value or set of different parameters. Furthermore, the applications of this acoustic filtering sensor to other industries are also an important research topic for the authors.

## Figures and Tables

**Table 1:** The structure parameters of the sensor.

Parameter Name	Nomenclature	Value	Units
The radius of cantilever plate	$a_{1}$	30	mm
The radius of strain region	$a_{2}$	7	mm
The velocity of sound in dimethylsilicone oil	$c_{0}$	1275	m/s
The density of dimethylsilicone oil	$\rho_{0}$	963	kg/m^3^
The thickness of cantilever plate	$h_{1}$	7	mm
The thickness of strain region	$h_{2}$	1	mm
The density of steel	ρ	7860	kg/m^3^
Young modulus of steel	E	206	Gpa
Poisson ratio of steel	μ	0.3	-

**Table 2:** The effect of different fluids on the coupling modal of the sensor.

Fluid Type	1st Order Frequency/Hz	2nd Order Frequency/Hz	3rd Order Frequency/Hz	4th Order Frequency/Hz
No fluid	1202.76	1381.92	20,444.3	28,787.2
Dimethylsilicone Oil	1202	2999	12,331	20,443
Water	1202	3268.88	14,113.1	20,444
Mercury	1202	4036	12,861.3	20,434
